# Supplementary material for: Reconfigurable optoelectronic transistors for multimodal recognition
Source: Nat Commun. 2024 Apr 16;15:3257. doi: 10.1038/s41467-024-47580-2 (PMC11021444; doi:10.1038/s41467-024-47580-2)
Supplement: Supplementary file 1 — Supplementary Information [file 41467_2024_47580_MOESM1_ESM.pdf]

Supplementary Information for

Reconfigurable optoelectronic transistors for multimodal recognition

Pengzhan Li[1,2#], Mingzhen Zhang[1,3#], Qingli Zhou[2#], Qinghua Zhang[1,4], Donggang Xie[1], Ge Li[1], Zhuohui Liu[1,5], Zheng Wang[1], Erjia Guo[1,3], Meng He[1], Can Wang[1,3], Lin Gu[6], Guozhen Yang[1], Kuijuan Jin[1,3*], and Chen Ge[1,3*]

[1] Beijing National Laboratory for Condensed Matter Physics, Institute of Physics, Chinese Academy of Sciences, Beijing 100190, China

[2] Key laboratory of Terahertz Optoelectronics, Ministry of Education, Department of Physics, Capital Normal University, Beijing 100049, China

[3] School of Physical Sciences, University of Chinese Academy of Science, Beijing 100049, China

[4] Yangtze River Delta Physics Research Center Co. Ltd., Liyang 213300, China

[5] College of Materials Science and Opto-Electronic Technology, University of Chinese Academy of Sciences, Beijing 100049, China

[6] Beijing National Center for Electron Microscopy and Laboratory of Advanced Materials, Department of Materials Science and Engineering, Tsinghua University, Beijing 100084, China

[#] These authors contributed equally: Pengzhan Li, Mingzhen Zhang, Qingli Zhou

* Correspondence and requests for materials should be addressed to K.J. (email: kjjin@iphy.ac.cn) or to C.G. (email: gechen@iphy.ac.cn).

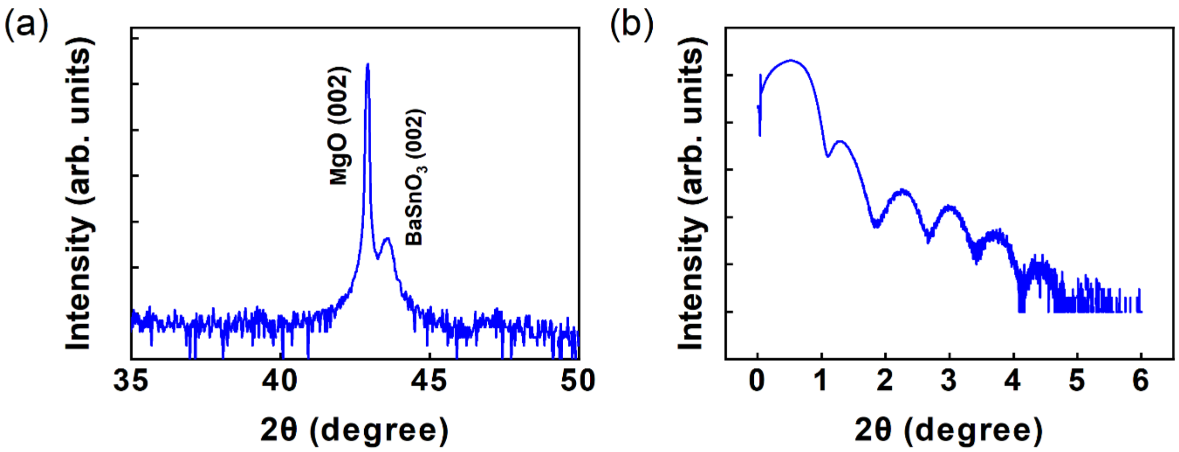

Supplementary Figure 1. Sample characterization of $BaSnO_3$ films. a, X-ray diffraction (XRD) $\theta - 2\theta$ scan of as grown $BaSnO_3$ thin film on (001)-oriented MgO substrate. **b,** X-ray reflectometry measurement result of $BaSnO_3$ film.

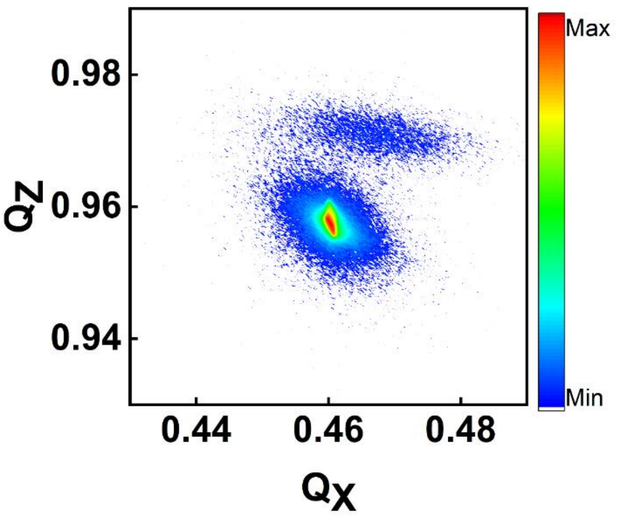

Supplementary Figure 2. Reciprocal space mapping around (204) reflections of $BaSnO_3$ film on (001)-MgO substrates.

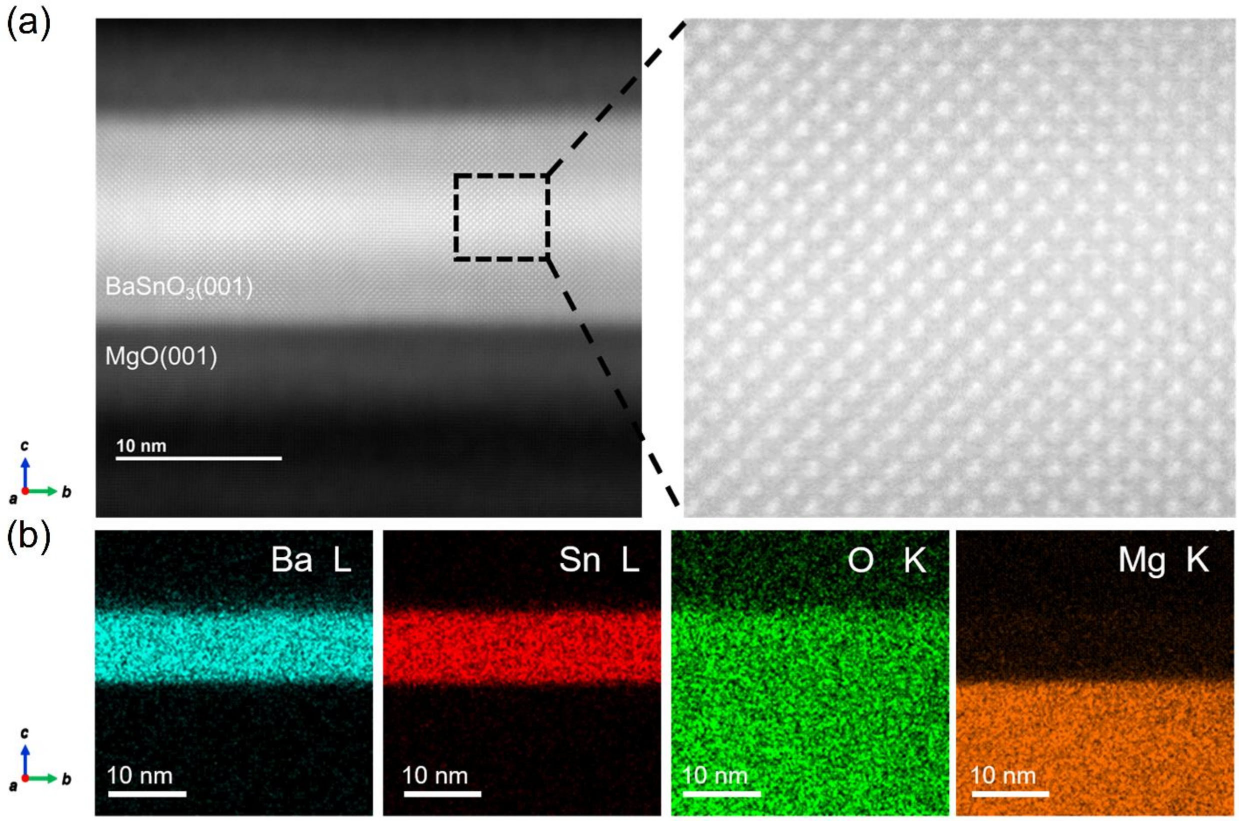

Supplementary Figure 3. STEM and EDS analysis of $BaSnO_3$ thin films a, High-angel annular dark-field scanning transmission electron microscopy image from a 10 nm thick $BaSnO_3$ film on MgO (001)**,** and the enlarged view of the selected area. **b**, Energy dispersive X-ray spectroscopy maps of the Ba La, Sn La, O K, and Mg k peaks. The scale bar is 10 nm.

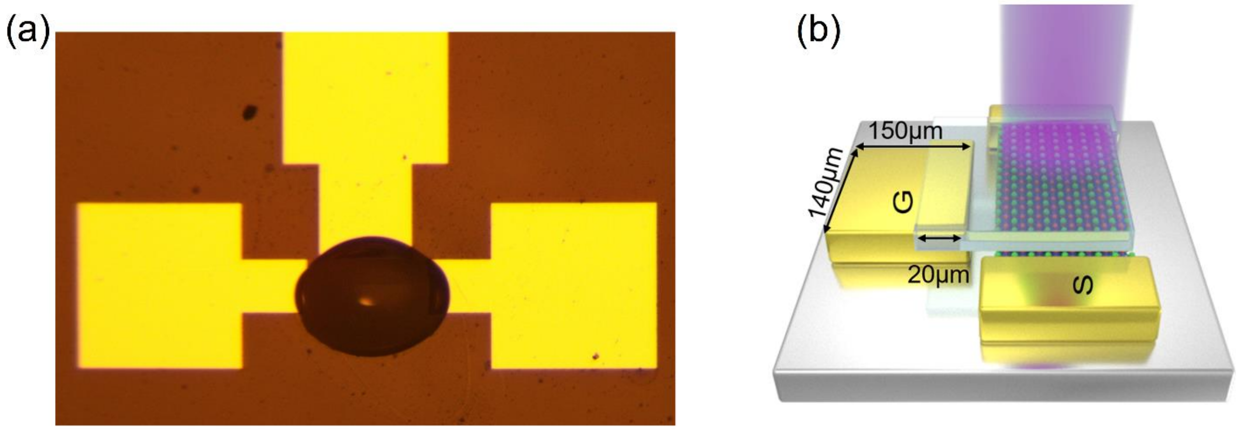

Supplementary Figure 4. Schematic diagram of device structure. a, Optical microscopy image of a coplanar (side-gate) BSO-EGT**. b,** the left view of the device structure.

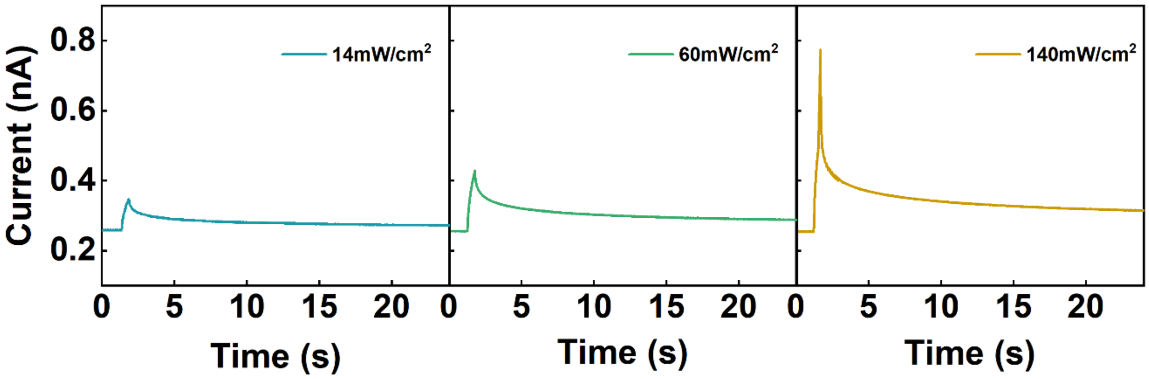

Supplementary Figure 5. The effect of UV light intensity on the channel current. Relaxation time of $BaSnO_3$ device under various light intensity with the same illumination durations 1s.

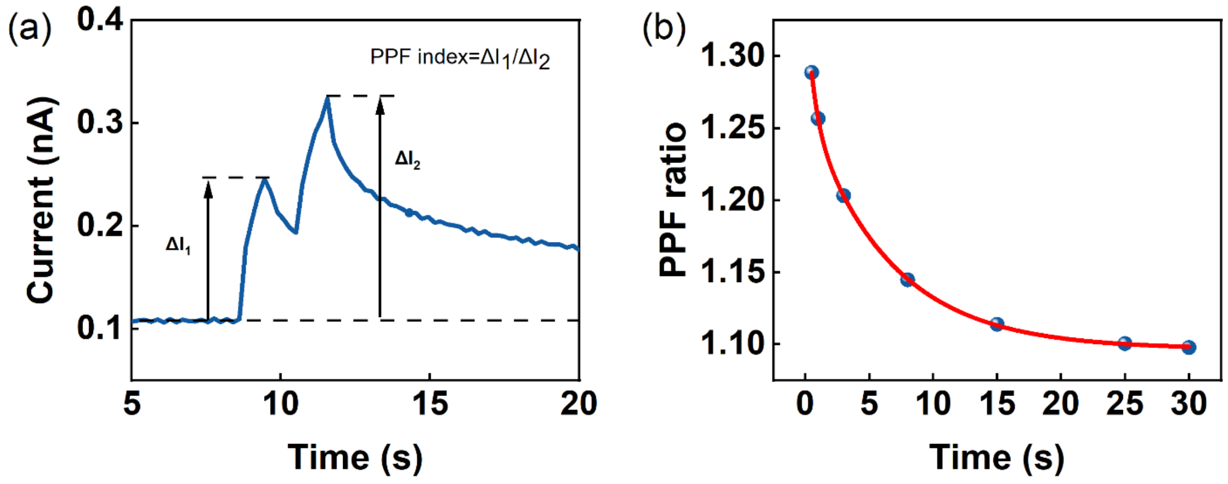

Supplementary Figure 6. Paired pulse facilitation (PPF) plasticity. a, Channel current is measured under UV irradiation with identical light intensity and duration time (light intensity of 70 mW/cm^2, the duration of 1 s). **b**, PPF ratio as a function of the pulse intervals, where the red line represents fitting results using the double exponential decay function.

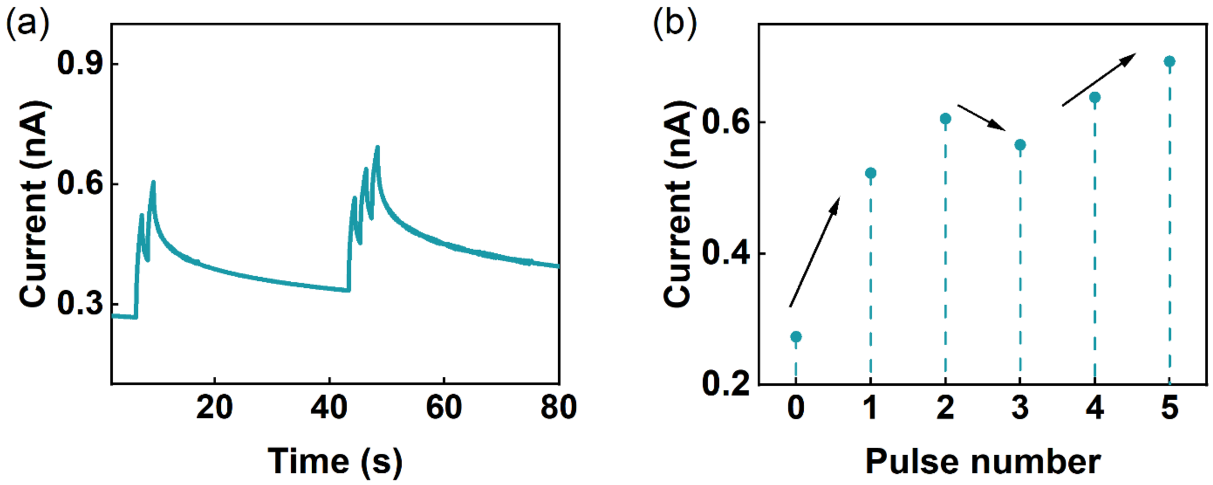

Supplementary Figure 7. Influence of pulse time interval on channel current. a, Changes of channel current stimulated by UV pulses (light intensity of 70 mW/cm^2, the duration of 1 s) with varying pulse intervals. **b,** Relationship between the channel current and the number of pulses, the current value after the pulse is applied for 1 s is used as the sampling point

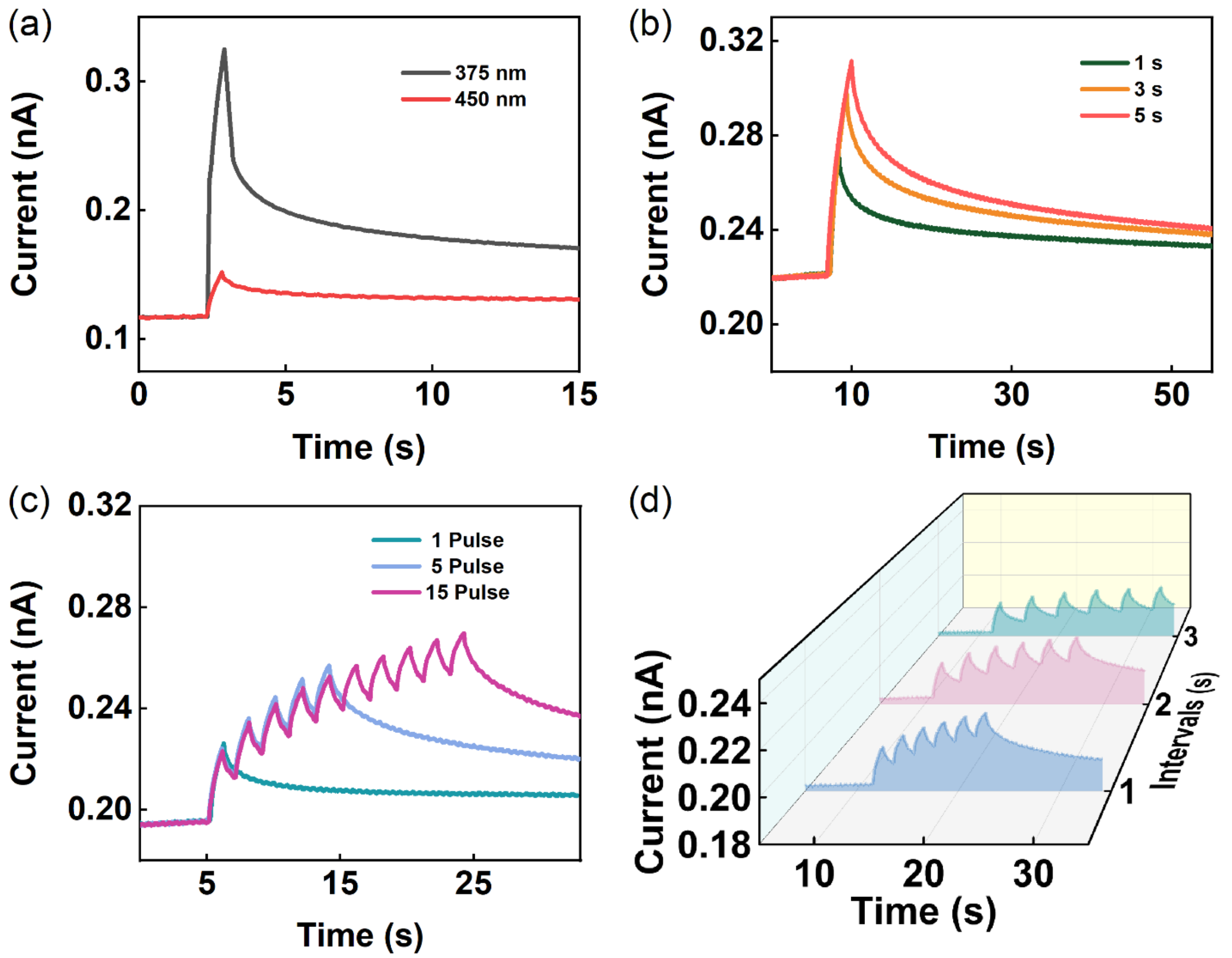

Supplementary Figure 8. The effect of the light exposure on the channel current under blue laser irradiation. a, Device response to illumination of different wavelengths (210 mW/cm^2, duration 1 s) under the same conditions. Among them, UV causes a more obvious change in conductance. **b,** I_{SD} response to blue light irradiation at different durations (140 mW/cm^2). **c**, Spike number dependent plasticity under blue light irradiation with the same pulse width and intervals (140 mW/cm^2 for 1 s). **d**, Relationship between channel current and sequence pulse interval, the channel current is measured under light irradiation with different interval duration (light intensity of 140 mW/cm^2, the duration of 1 s)

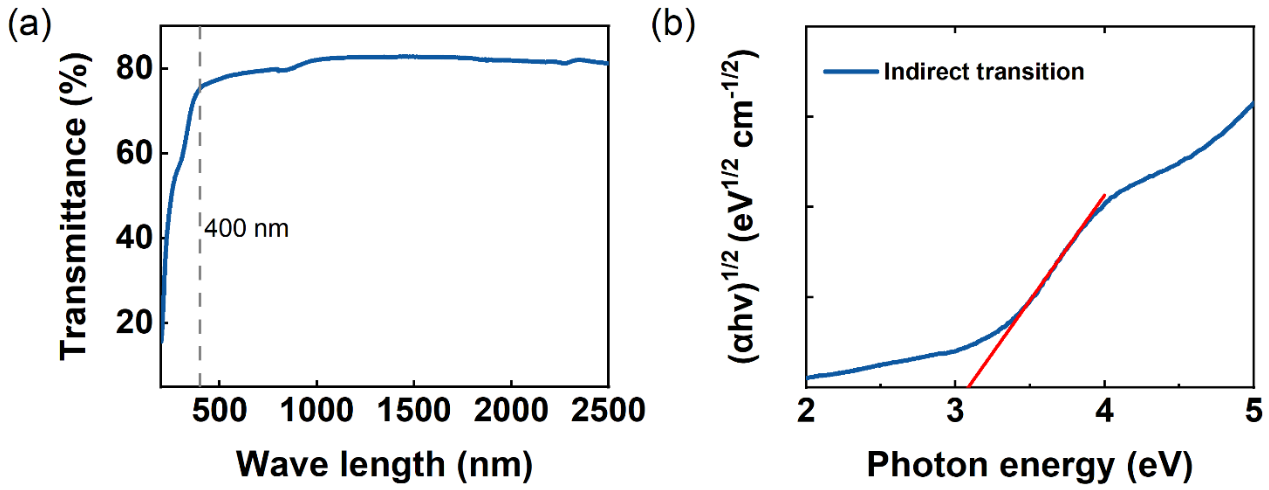

Supplementary Figure 9. Optical transmittance spectra of $BaSnO_3$. a, Optical transmittance spectrum of 20-nm-thick $BaSnO_3$ film at UV-Vis-NIR wavelength. **b**, Tacu plot for indirect transition to estimate band gap. The $BaSnO_3$ films is transparent at visible and near-infrared wavelength (i.e., $\lambda >$ 400 nm) due to the large band gap (E_g= 3.1 eV).

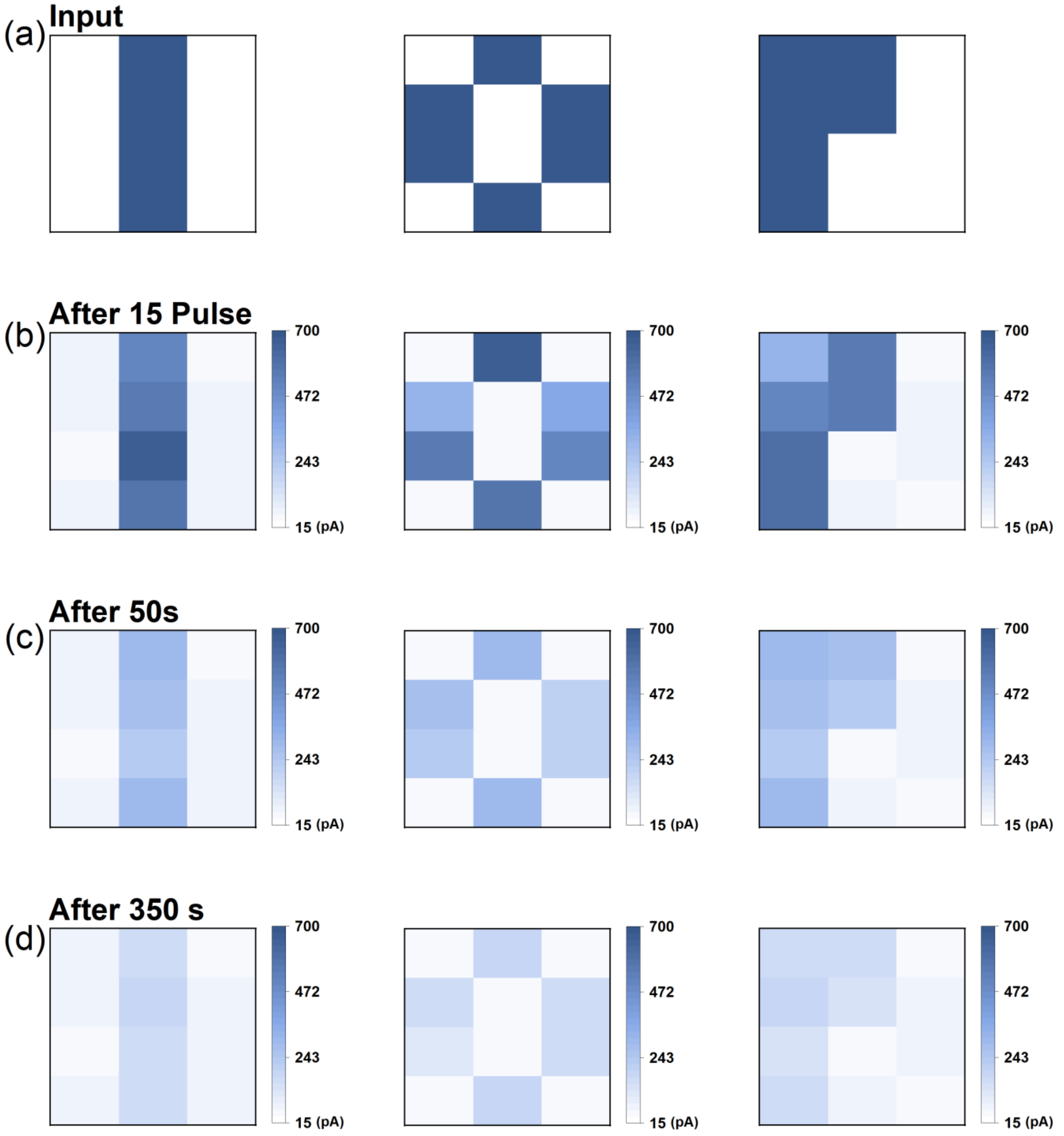

Supplementary Figure 10. A 3×4 array for pattern learning. a, Three letters "I", "O" and "P" are selected as inputs to the array. **b,** The device current (I_{SD}) was recorded after 15 UV pulse irradiations (70 mW/cm^2, duration 2 s). **c,** Channel current distribution at 50 s after the end of the last light pulse. **d,** Channel current distribution at 350 s after the end of the last light pulse.

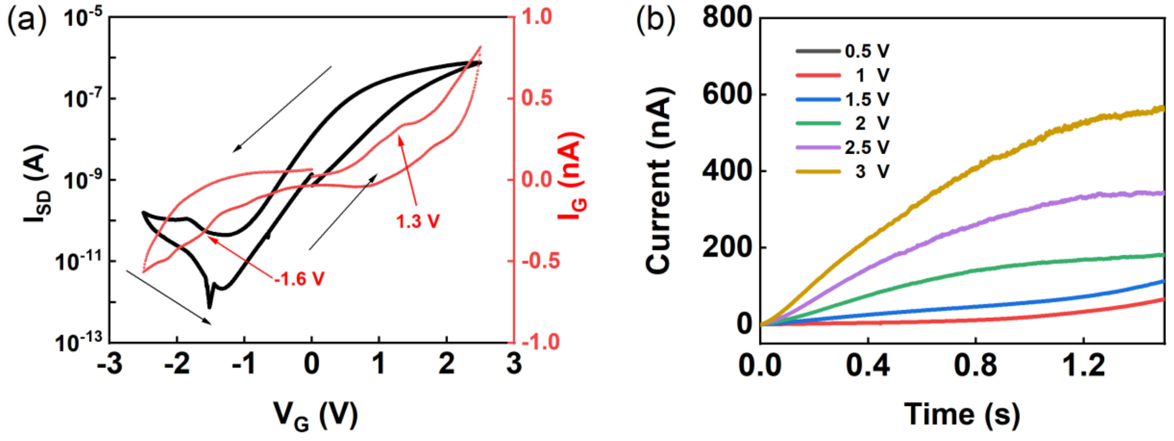

Supplementary Figure 11. Electrical performance of $BaSnO_3$ transistor. a, Transfer curve of the $BaSnO_3$ electrolyte-gated transistor (BSO-EGT) measured on V_{SD}=0.5 V. The gate voltage was swept from 0 V to 2.5 V, 2.5 V to -2.5 V, and then back to 0 V. **b,** Output curve of BSO-EGT at a V_{DS} range of 0 to 1.5V.

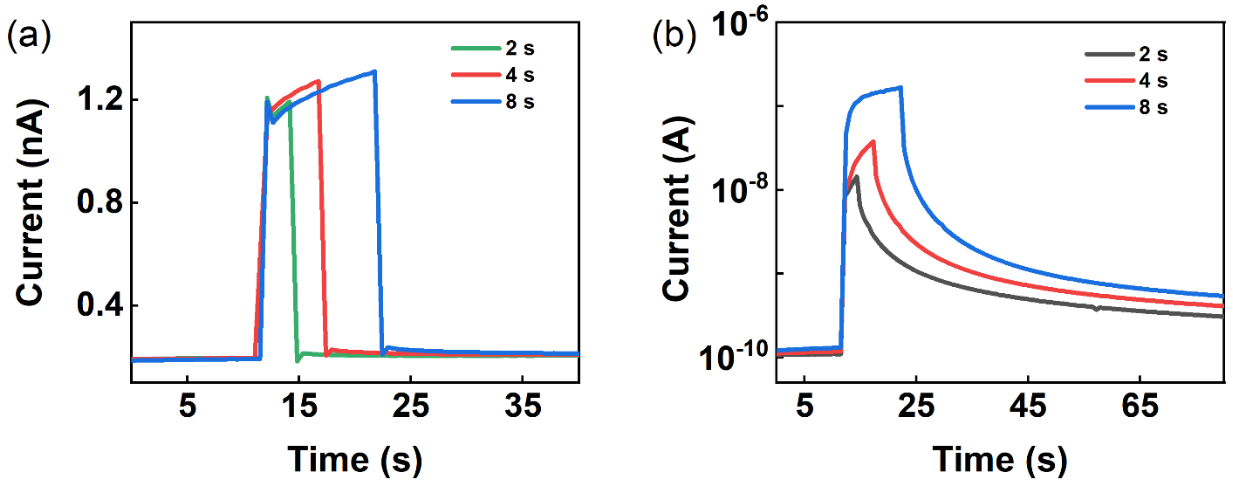

Supplementary Figure 12. Long-term and short-term plasticity of $BaSnO_3$ transistors. a, Under the voltage stimulation of V_G=1 V (with different durations), the channel current can quickly return to the initial state. **b**, Under the electrical pulse stimulation of V_G=2 V, the conductance of the device exhibits obvious non-volatile characteristics.

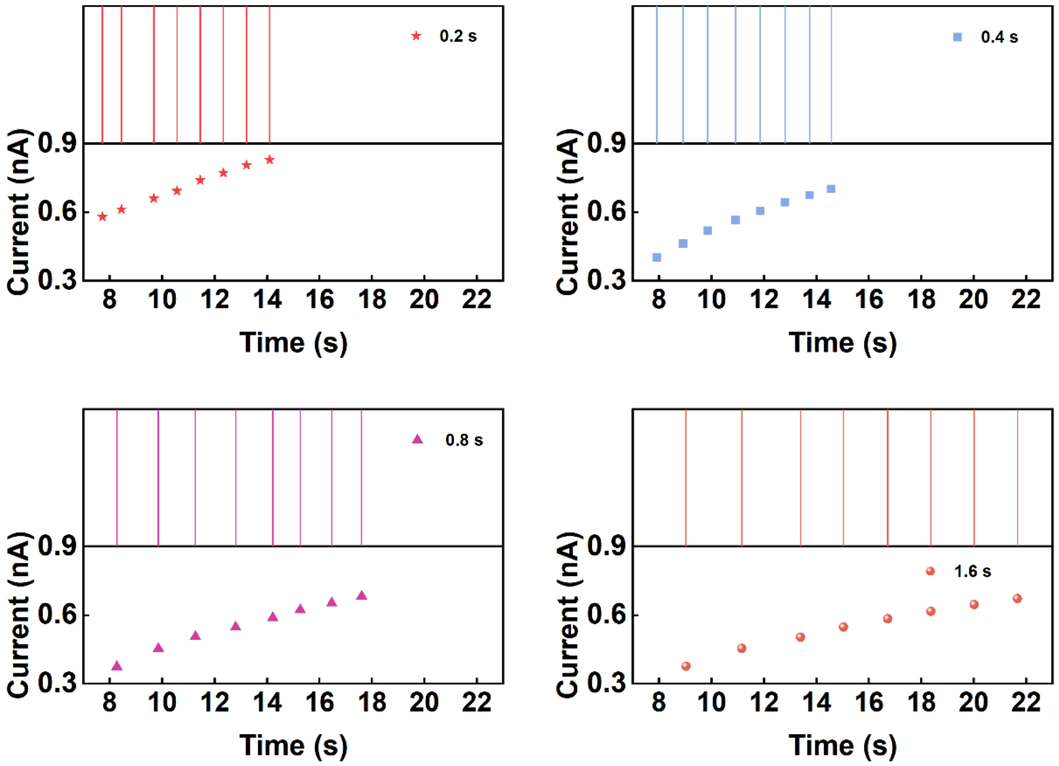

Supplementary Figure 13. Spike-frequency-dependent plasticity. Current change subject to 8 voltage pulse (V_G=1 V, 1 s) with different pulse intervals.

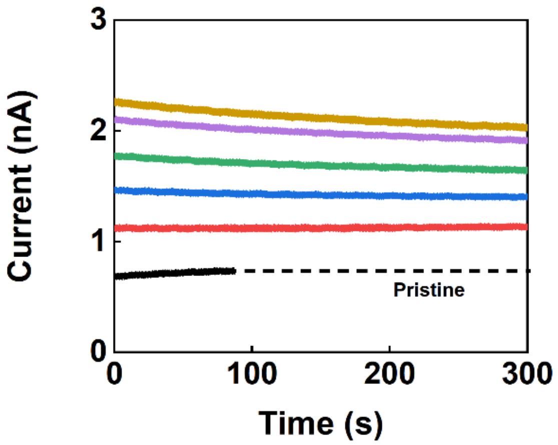

Supplementary Figure 14. The non-volatile multi-level conductance retention properties. The multi-states are produced by a series of V_G pulses with different durations (V_G= +2 V, durations from 1 s to 5 s).

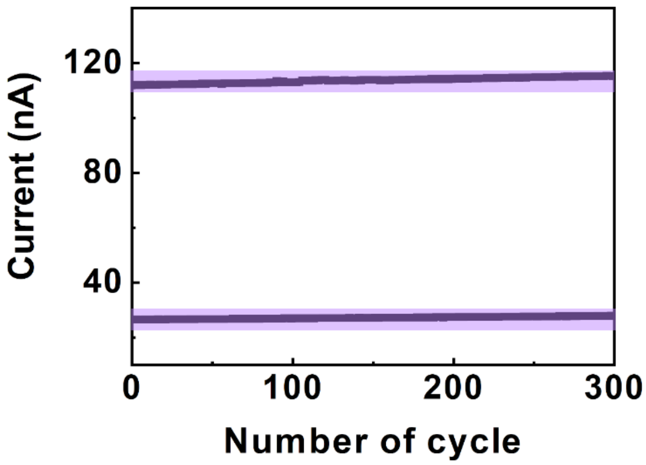

Supplementary Figure 15. Pulse-switching characteristics. The high conductance state is generated by V_G= 2 V, and the low conductance state is generated by V_G= -2 V. The pulse duration is 1 s. We choose 1 s after applying the voltage pulse stimulation as the sampling point.

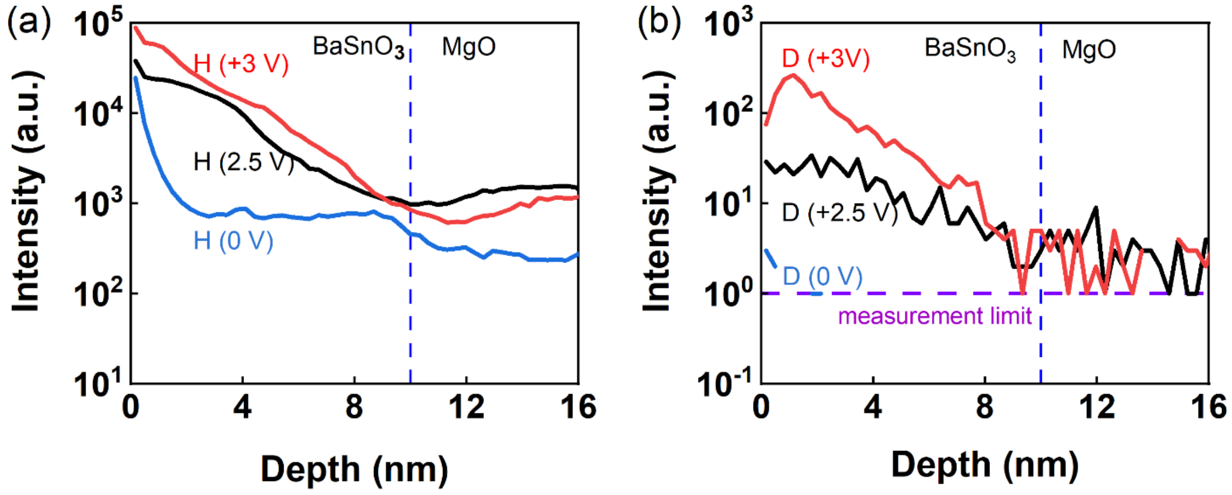

Supplementary Figure 16. Secondary-ion mass spectrometry (SIMS) depth profiling in the pristine and gated $BaSnO_3$ films. a, Penetration depth of H^+ ions as the voltage increases, the content of H^+ ions is getting higher and higher. Ion migration mechanism demonstrated during positive voltage application. Here, the gating time is 5 hours. **b,** The penetration depth of D^+ ions. The $BaSnO_3$ films were gated with the ionic liquids containing heavy water D_2O, No D^+ signal detected in the pristine film without electrolyte conditioning.

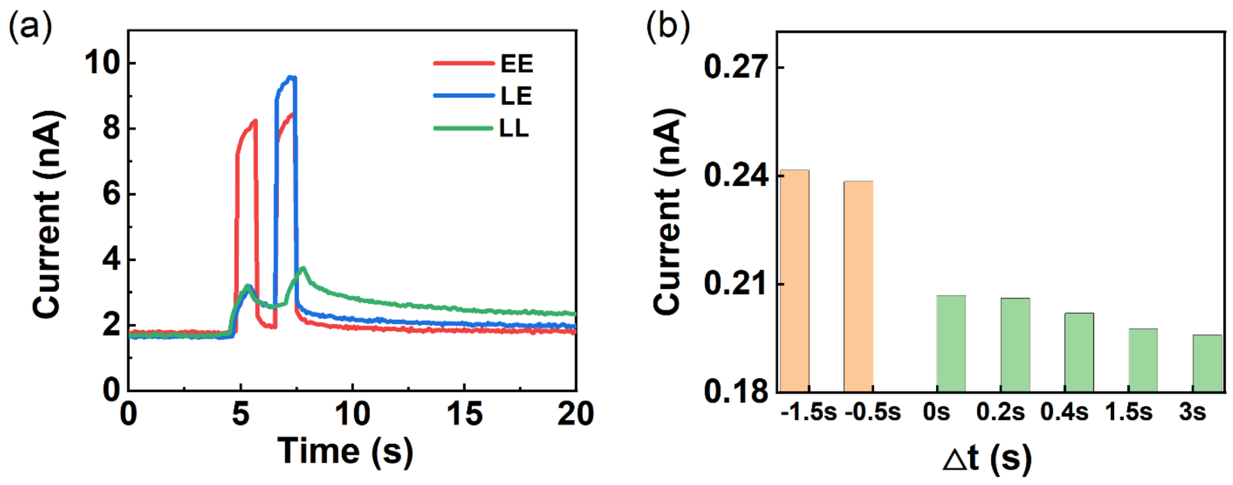

Supplementary Figure 17. Device Characteristics for Mixed-Mode Input. a, Changes in channel current are monitored by a 0.3 V read voltage at the three mode inputs ("LL", "LE", "EE"). Among them, "LL" represents two optical pulses (70 mW/cm^2), "LE" represents one optical pulse and one electric pulse, and "EE" represents two electric pulse signal inputs (1 V). The pulse width and time interval are both 1 s. **b**, Under "LE" mode input, the influence of the time interval between two pulses on the current value of the sampling point, the current value is collected 1 s after the second pulse is applied.

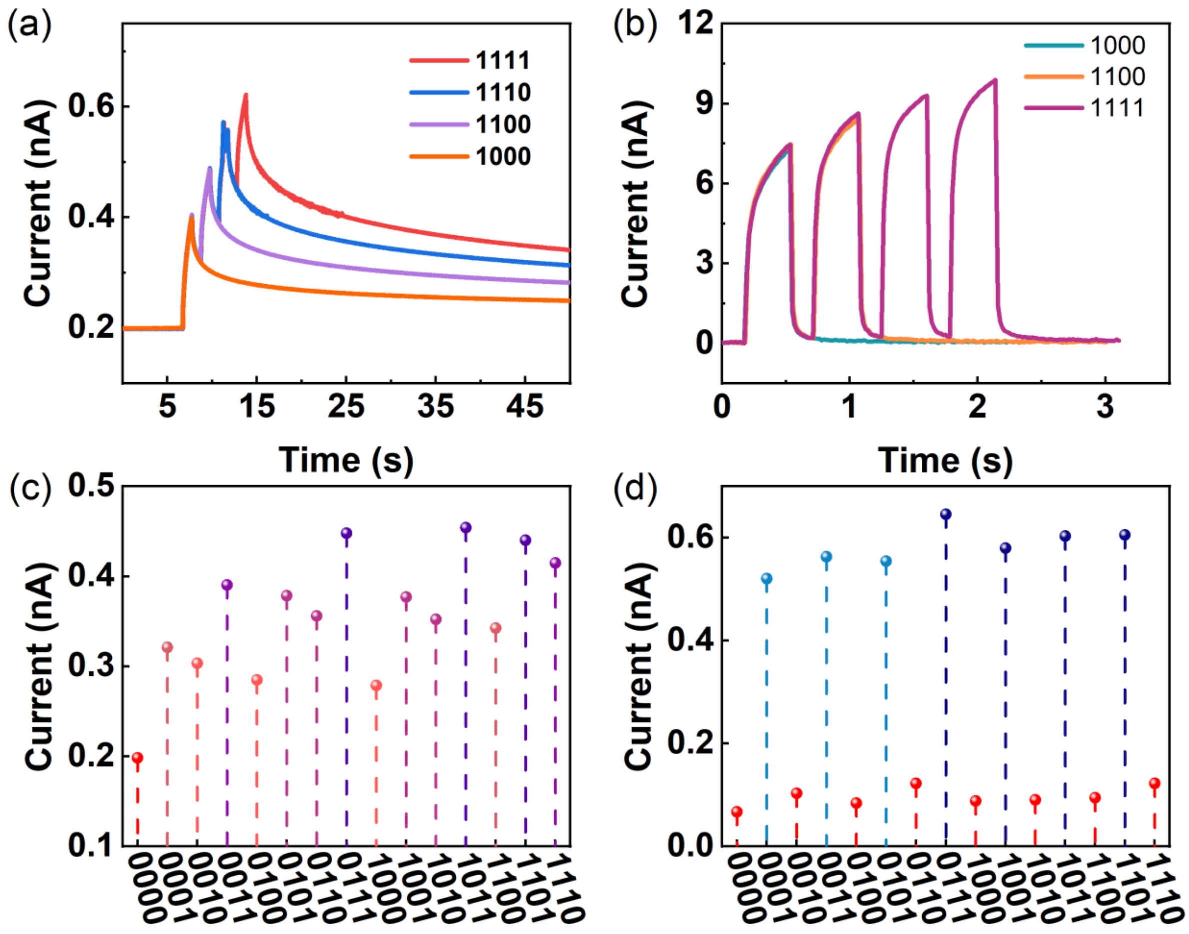

Supplementary Figure 18. Nonlinear mapping of 4-bit inputs based on the BSO reservoir. a, b, Changes in channel currents in response to light and electrical stimulation with different coding combinations, respectively. **c, d,** show the final distinguishability of the 16 encodings under light and electrical stimulation, respectively. The final distinguishable state is taken at 1 s after the application of the fourth pulse.

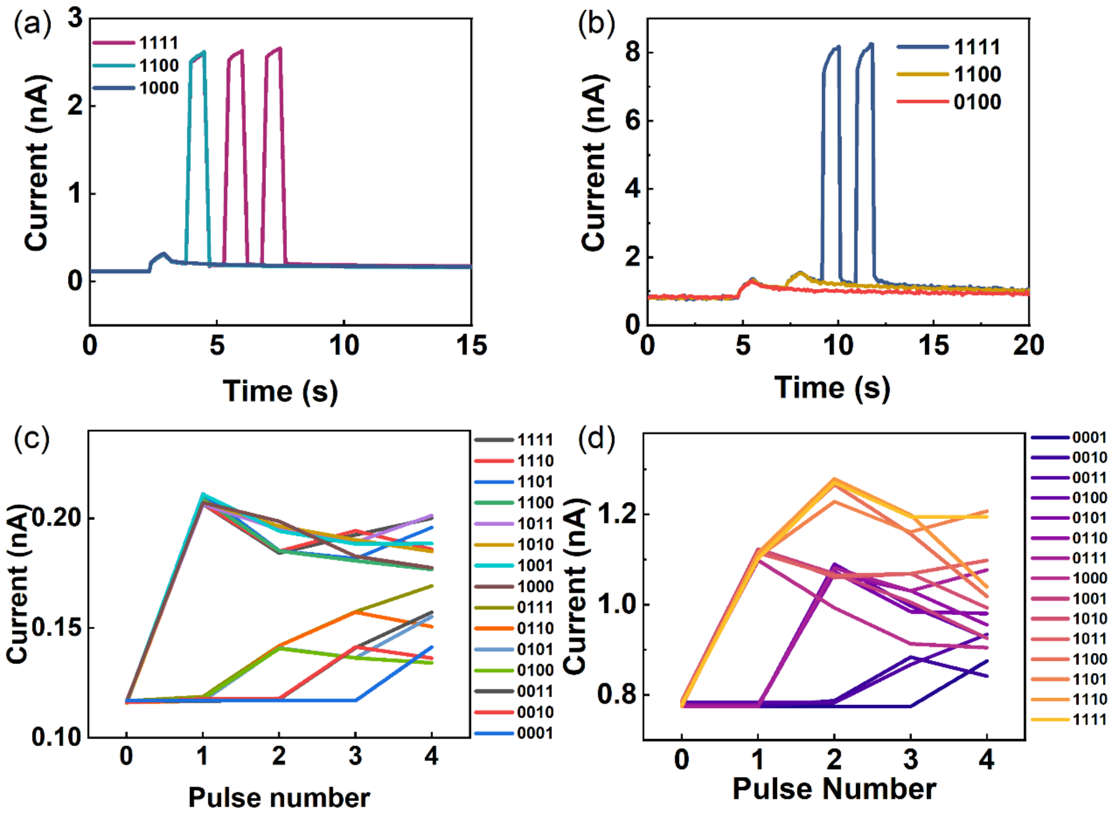

Supplementary Figure 19. Multimodal nonlinear dynamics for reservoir computing. Variety of input waveform patterns. **a,** "LEEE" mode, **b,** "LLEE" mode demonstrate different current states distribution. **c,** The distinguishable output of 4-bit reservoir states of "LEEE" input. **d,** The distinguishable output of 4-bit reservoir states of "LLEE" input.

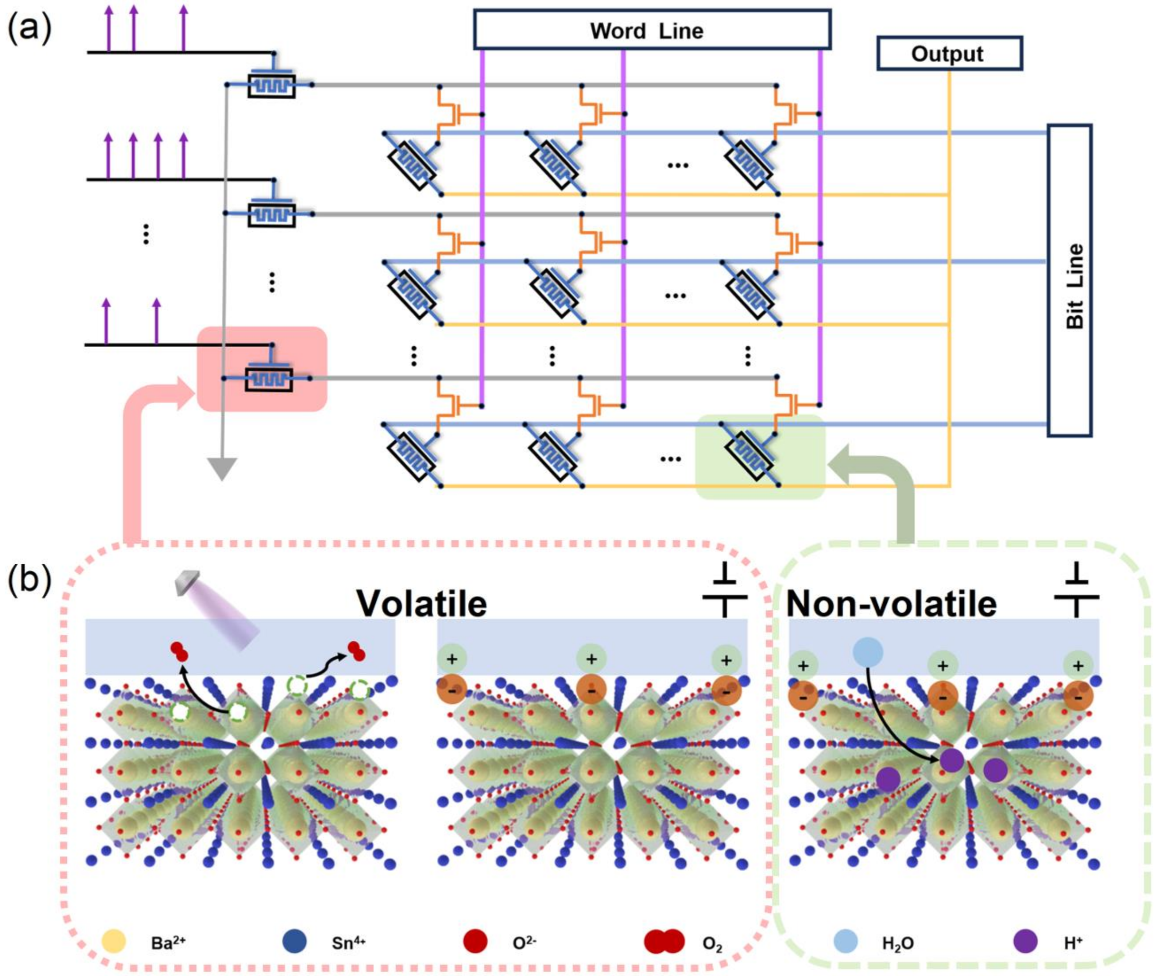

Supplementary Figure 20. System structure diagram of realizing reservoir and ANN functions based on BSO-EGT. a, Network diagram designed for hardware implementation of BSO-EGT based reservoir computing and artificial neural network. **b**, The volatility exhibited by the device stems from two physical mechanisms. One is the generation of oxygen vacancies locally on the surface of the film caused by UV light. The other one comes from the charge accumulation induced by the electric double layer effect during low voltage regulation. The non-volatility of the device comes from the ion migration during hydrolysis under high voltage.

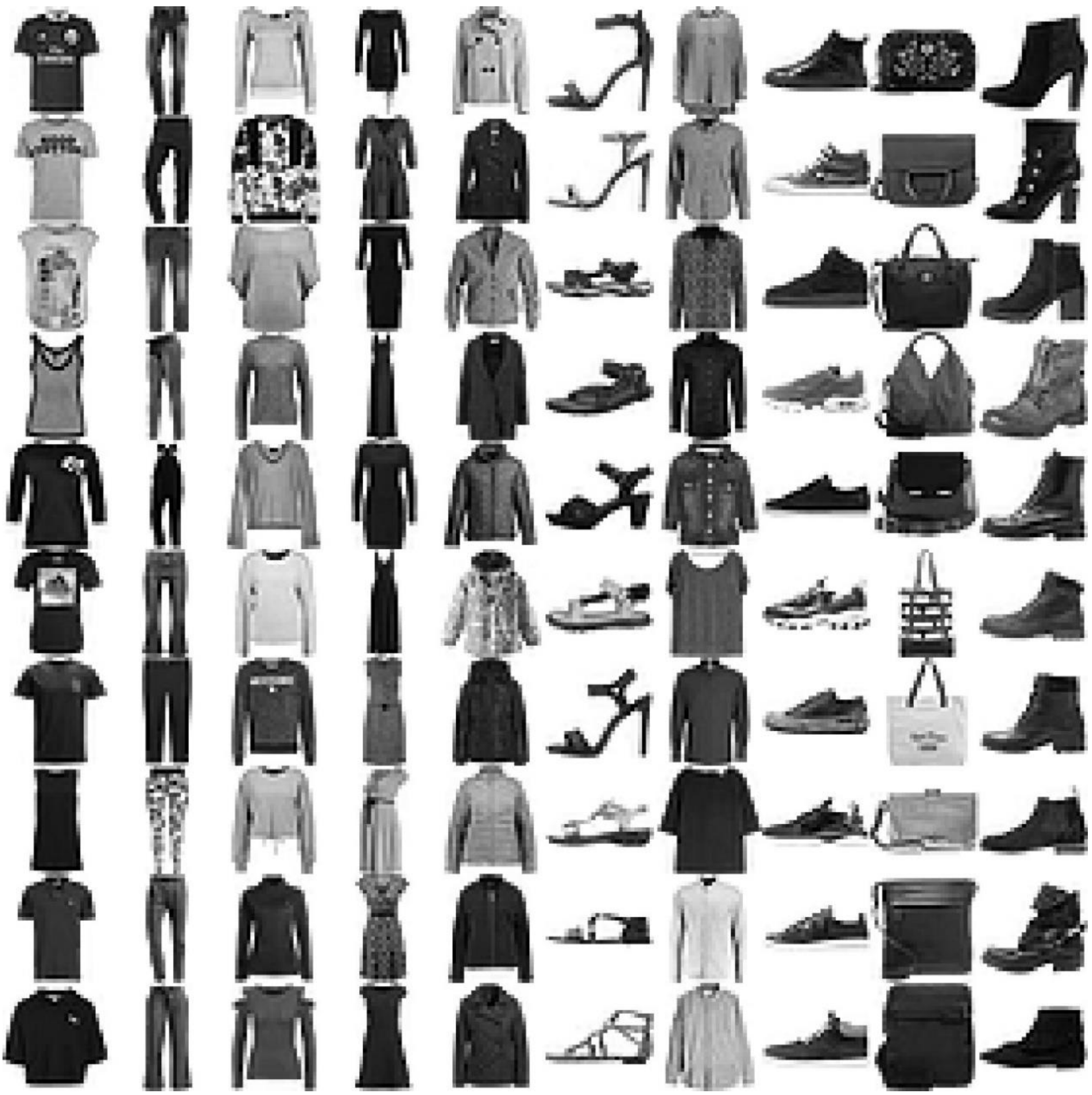

Supplementary Figure 21. Schematic representation of the Fashion-MNIST dataset[1] used in the recognition task. We categorized these images into 5 categories of tops, pants, dresses, shoes and bags.

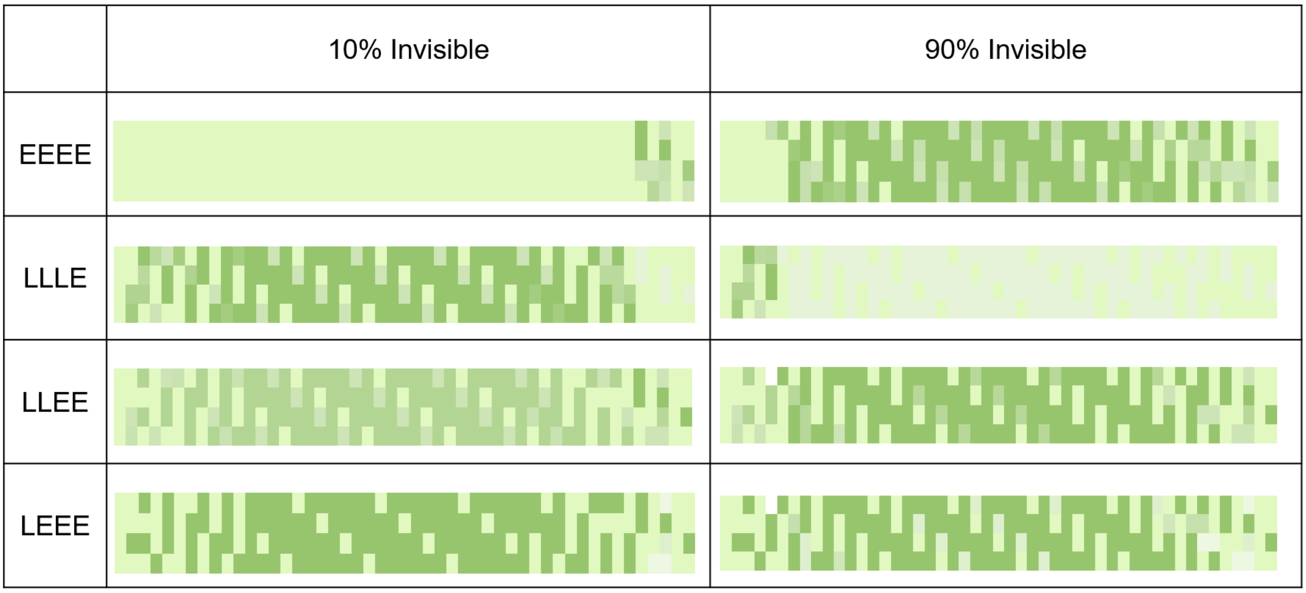

Supplementary Figure 22. outputs of the “EEEE”, “LLLE”, “LLEE” and “LEEE” reservoirs with invisible degree of 10% (left panel) and 90% (right panel), respectively.

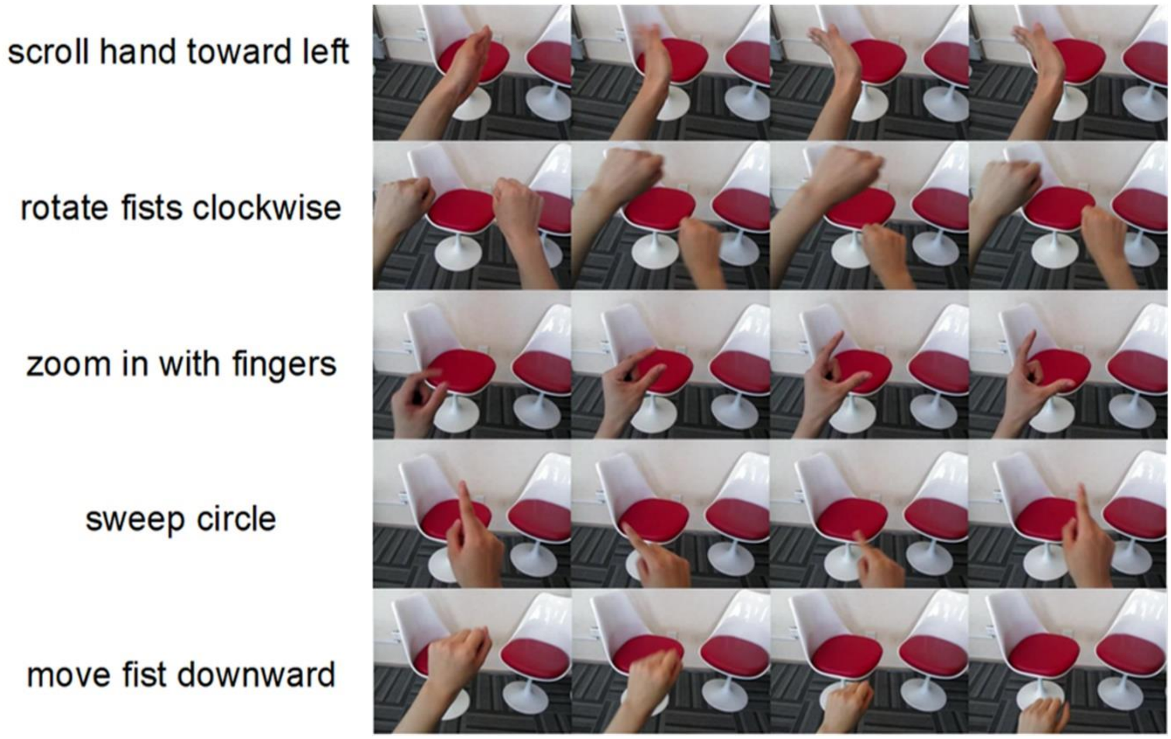

Supplementary Figure 23. Schematic representation of the Ego-Gesture dataset used in the recognition task. Five different kinds of actions were selected for recognition in the audiovisual fusion task. The voice input corresponding to each action is generated by AI.

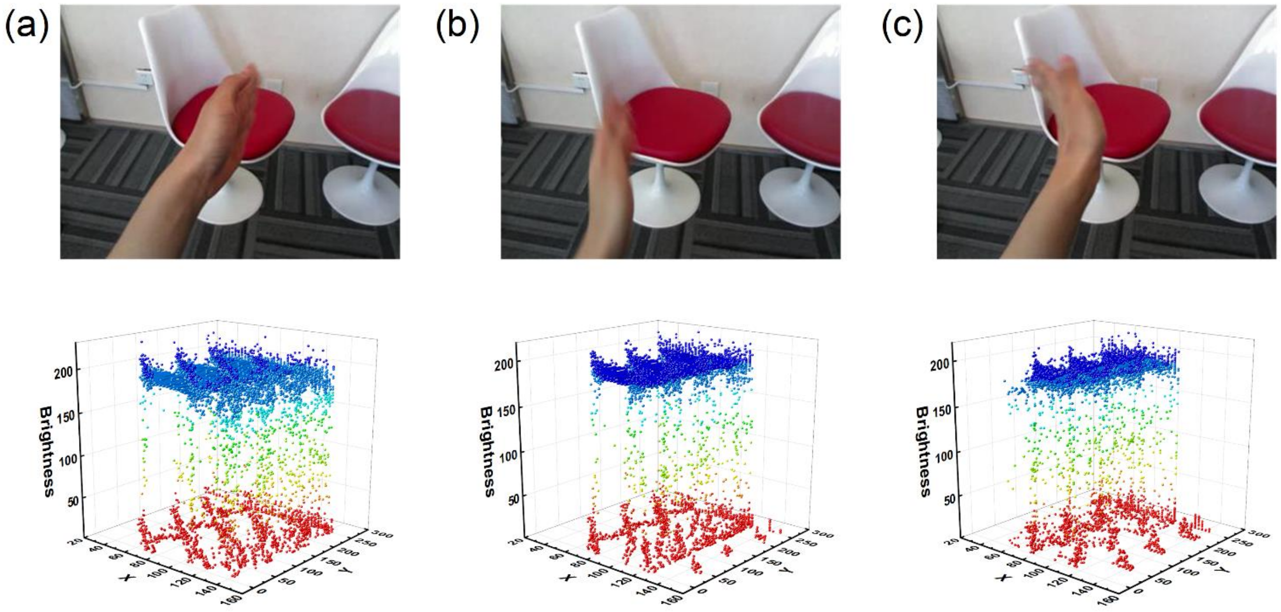

Supplementary Figure 24. Three-dimensional spatial mapping of the hand gesture for recognition. a-c, Each of the three frames corresponds to the actual movement of the hand at different times, and the lower part corresponds to the three-dimensional mapping of the movement.

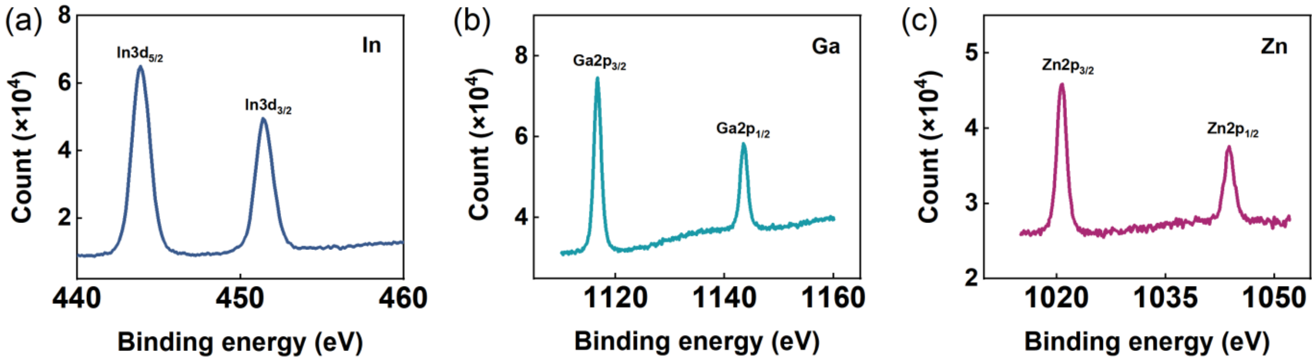

Supplementary Figure 25. X-ray photoelectron spectroscopy (XPS) spectra of a, In 3*d*, **b,** Ga 2*p* and **c,** Zn 2*p* in an IGZO thin-film.

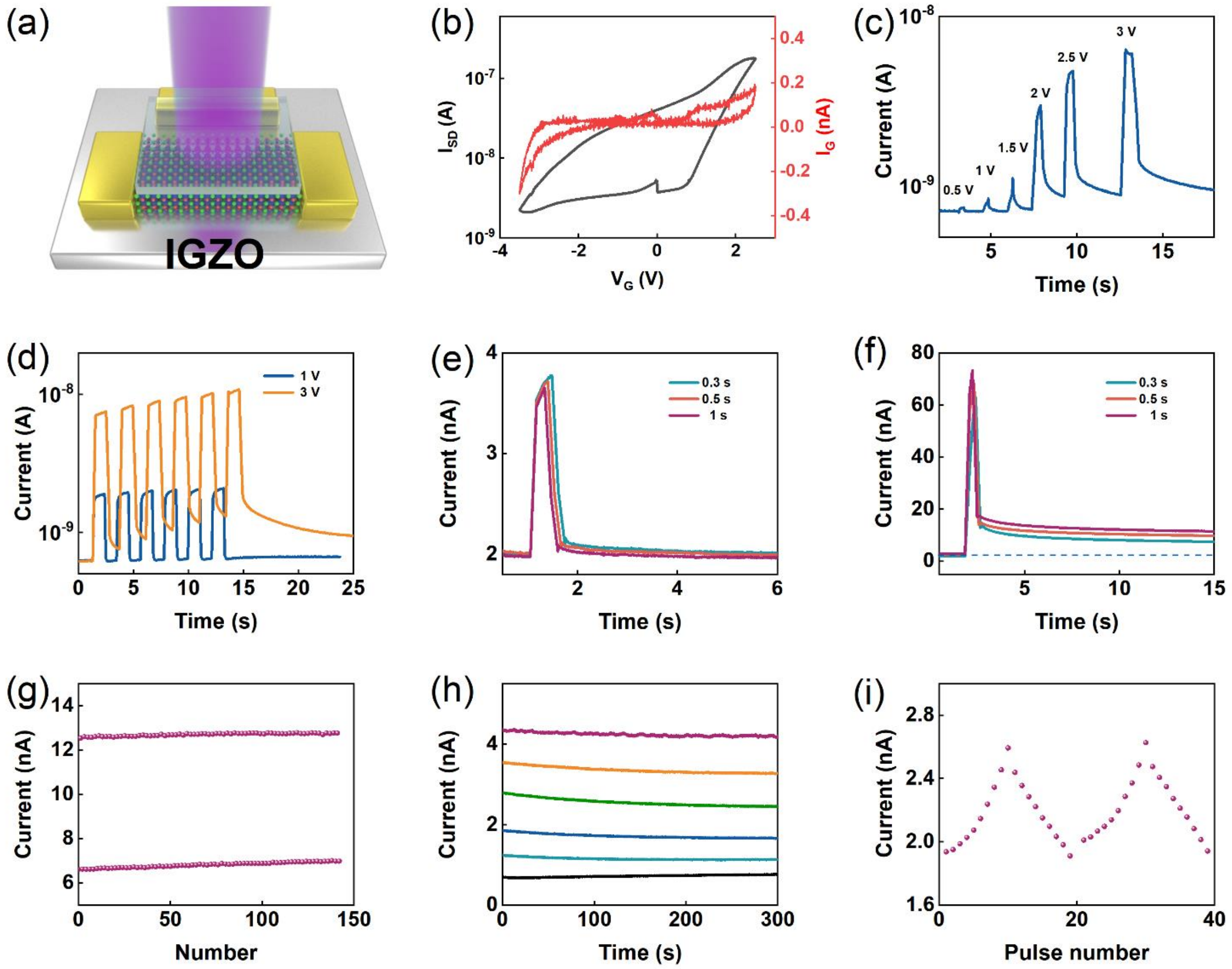

Supplementary Figure 26. Electrical response characteristics of IGZO-EGT. a, Schematic illustration of the neuromorphic transistor that can be stimulated using optical and electrical signals. The $InGaZnO_4$ film serves as a channel between the source (S) and drain (D) electrodes, and IL is used as the gating medium. **b,** Transfer curves of the IGZO transistor measured with V_{SD} = 0.5V. **c,** The channel current controlled through a series of V_G pulse with a pulse width of 1s and different amplitudes which shows the transition of the PSC from volatile to non-volatile property. **d,** Channel current under voltage stimulation of +1 V and +3 V. **e,** +1 V voltage pulse stimulation with different pulse durations. **f,** +2.5 V voltage pulse stimulation with different pulse durations. **g,** Pulse-switching characteristics. The high conductance state is generated by V_G= +2.5 V, and the low conductance state is generated by V_G= -2.5 V. **h,** The non-volatile multi-level conductance retention properties. The multi-states are produced by a series of V_G pulses with different pulse numbers (V_G= +2.5 V). **i,** Cyclic controlled LTP using V_G (equally spaced from +1.5 to +3.5 V, duration of 1 s, spaced 1 s apart) and LTD using V_G (equally spaced from -1.5 to -3.5 V, duration of 1 s, spaced 1 s apart) for 10 pulses.

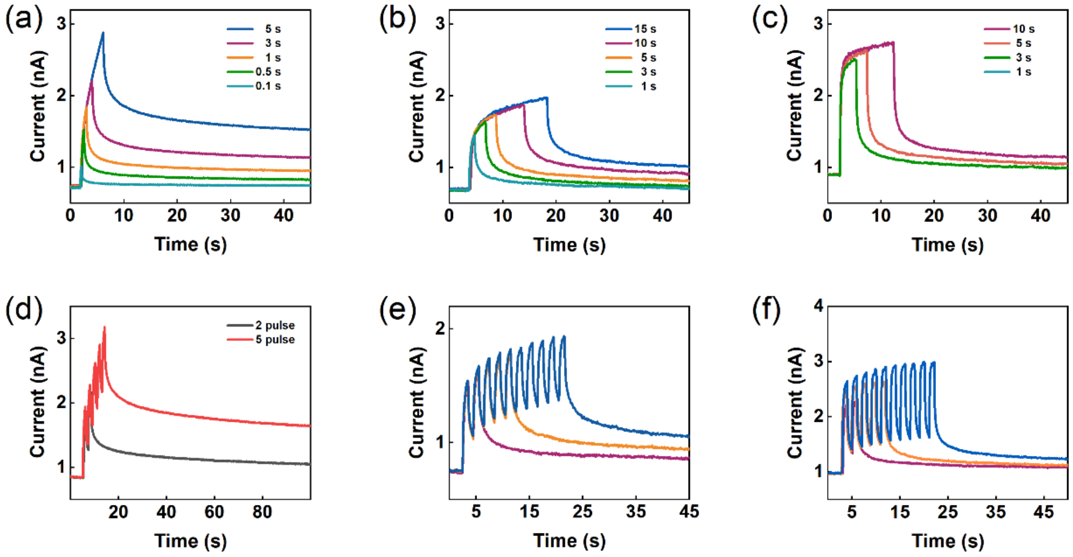

Supplementary Figure 27. Photoresponse characteristics of IGZO-EGT. a, Evolution of channel current under UV light (375 nm) irradiation of different durations (7 mW/cm^2). **b,** Evolution of channel current under blue light (450 nm) irradiation of different durations (70 mW/cm^2). **c,** Evolution of channel current under green light (532 nm) irradiation of different durations (70 mW/cm^2). **d-f**, The evolution of channel current stimulated by different numbers of light pulses, respectively under UV (375nm), blue (450nm) and green light (532nm) illumination.

Ref.	On/off ratio	Optical reconfigurability	Electrical reconfigurability	Reservoir+Synapse	Multisensory integration	Task type
2	> 10^3	/	Yes	Yes	/	static
3	> 10^6	Potentially Yes	Yes	/	Tactile + Visual	static
4	> 10^6	/	Yes	Yes	/	Dynamic
5	> 10^3	/	/	/	Ocular+Vestibular	Dynamic
This work	> 10^6	Yes	Yes	Yes	Audio + Visual	Dynamic

Supplementary Table 1. Comparison with state-of-the-art neuromorphic transistors and systems

Supplementary References:

1. Xiao, H. *et al*. Fashion-MNIST: a novel image dataset for benchmarking machine learning algorithms. arXiv. https://arxiv.org/abs/1708.07747 (2017).

2. John, R. A. *et al*. Reconfigurable halide perovskite nanocrystal memristors for neuromorphic computing. *Nature Communications* 13, 2074 (2022).

3. Liu, K. *et al*. An optoelectronic synapse based on α-In_2Se_3 with controllable temporal dynamics for multimode and multiscale reservoir computing. *Nature Electronics* 5, 761-773 (2022).

4. Wang, S. *et al*. An organic electrochemical transistor for multi-modal sensing, memory and processing. *Nature Electronics* 6, 281-291 (2023).

5. Jiang, C. *et al*. Mammalian-brain-inspired neuromorphic motion-cognition nerve achieves cross-modal perceptual enhancement. *Nature Communications* 14, 1344 (2023).
